# Only three cycles of nivolumab showed remarkable durable response and reversible myeloradiculoneuropathy in a patient with metastatic papillary renal cell carcinoma type 2

**DOI:** 10.1002/iju5.12262

**Published:** 2021-02-17

**Authors:** Kazunori Iwasaki, Toshitaka Shin, Toru Inoue, Tadamasa Shibuya, Kenichi Hirai, Tadasuke Ando, Hiromitsu Mimata

**Affiliations:** ^1^ Department of Urology Oita University Faculty of Medicine Yufu Oita Japan

**Keywords:** durable response, immune‐related adverse event, myeloradiculoneuropathy, nivolumab, papillary renal cell carcinoma

## Abstract

**Introduction:**

The efficacy of nivolumab for non‐clear cell renal cell carcinoma is still unclear. We present a rare case of metastatic papillary renal cell carcinoma remarkably responded to nivolumab but developed myeloradiculoneuropathy as immune‐related adverse event.

**Case presentation:**

The patient had previously undergone radical nephrectomy for right renal mass and was diagnosed as papillary renal cell carcinoma type 2, pT3bN0M0. Three years after the first surgery, he received 3 mg/kg of nivolumab as a second‐line drug for mediastinum lymph nodes and lung metastases. With three cycles of nivolumab, the patient felt progressive weakness of the legs and received two cycles of steroid‐pulse therapy based on the diagnosis of myeloradiculoneuropathy. Although nivolumab therapy has been discontinued, the metastases show radiographic complete response at 2 years after the last nivolumab administration without any additional therapy.

**Conclusion:**

Nivolumab may be a promising treatment option for non‐clear cell renal cell carcinoma such as papillary renal cell carcinoma.

**Abbreviations & Acronyms**
ccRCC: clear cell renal cell carcinoma
CR: complete response
ICI: immune checkpoint inhibitor
IMDC: International Metastatic Renal Cell Carcinoma Database Consortium
irAE: immune‐related adverse event
PRCC: papillary renal cell carcinoma
RCC: renal cell carcinoma

**Keynote message**

We report a case of metastatic PRCC type 2 remarkably responded to only three cycles of nivolumab therapy but developed myeloradiculoneuropathy as irAE.

## Introduction

Immunotherapy with nivolumab, a fully human monoclonal immunoglobulin G4 programmed death 1 ICI antibody, has been approved for advanced and metastatic RCC as a second‐line systematic therapy since 2016 in Japan.^1^ On many guidelines around the world,^1^, ^2^, ^3^, ^4^ nivolumab is recommended as a treatment option for patients previously treated with antiangiogenic drugs such as tyrosine kinase inhibitors especially for ccRCC. It is based on CheckMate 025 phase III clinical trial, demonstrated nivolumab had overall survival benefit compared with everolimus for RCC with clear cell component.^5^ However, the efficacy of nivolumab for non‐ccRCC is still unclear. Herein we present a rare case of metastatic PRCC remarkably responded to nivolumab but developed myeloradiculoneuropathy as irAE.

## Case presentation

A 73‐year‐old man was referred to our hospital for the second‐line systematic treatment for metastatic PRCC involving mediastinum lymph nodes and lung metastases.

**Fig. 1:**
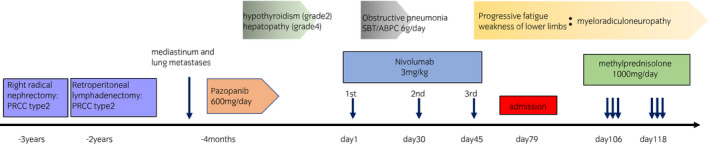
Progress chart for this case is shown. Myeloradiculoneuropathy was developed after three cycles of nivolumab administration, and treated with two cycles of steroid‐pulse therapy.

He had undergone a right radical nephrectomy 3 years before and was diagnosed with PRCC type 2, pT3bN0M0 (Fig. 1). The first recurrence appeared as a retroperitoneal lymph node metastasis 2 years before, and the lymph node resection was performed. The pathology confirmed metastatic PRCC. The second recurrence appeared as mediastinum lymph nodes and lung metastases 4 months before. Thus, the patient received first‐line pazopanib 600 mg/day once daily for several weeks, but hypothyroidism (grade 2) and drug‐induced hepatopathy (grade 4) occurred as adverse event and metastatic lesions increased on computed tomography. Based on the course of treatment so far, we chose to start immune checkpoint blockade as the second‐line systematic therapy with nivolumab at 3 mg/kg per body weight every 2 weeks intravenously.

The patient’s past medical history included diabetes mellitus and renal calculi. Medications included levothyroxine sodium hydrate, glimepiride, and alogliptin benzoate. His activities of daily living was not limited (Karnofsky Performance Status 100%). IMDC risk classification was a favorable risk at the time of starting the first‐line pazopanib therapy. Even before nivolumab therapy, the risk category of the modified IMDC criteria was a favorable risk.^6^

**Fig. 2:**
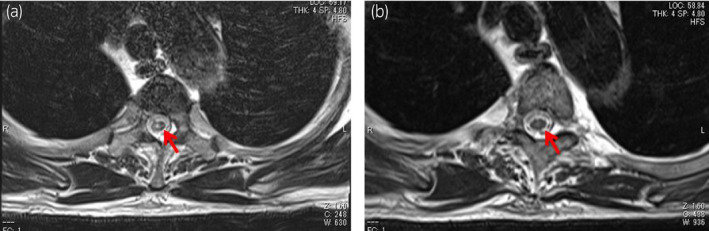
Spinal magnetic resonance imaging showed T2WI hyperintensities below Th2 levels (arrow) disappeared after two cycles of steroid‐pulse therapy. (a) Onset of myeloradiculoneuropathy. (b) After two cycles of steroid‐pulse therapy.

At the time of treatment on day 1, he had no significant symptoms. On day 7, obstructive pneumonia occurred because of increasing mediastinum lymph nodes and treated with sulbactam/ampicillin 6 g/day twice daily for 2 weeks. He received second and third dose of nivolumab on days 30 and 45, respectively. After the third nivolumab administration, the patient began to feel weakness of lower limbs and the symptoms were getting worse. On day 79, he was hospitalized due to the difficulty in walking by himself. Physical examination showed disturbance of lower limb deep sensibility and weakness of lower limb proximal muscle. Cerebrospinal fluid had good clarity, normal glucose level (95 mg/dL), increased lymphocyte‐dominated cell number (107/mm^3^; lymphocyte 99%), increased protein level (253.4 mg/dL), increased IgG level (20.4 mg/dL), and increased myelin basic protein (261.2 pg/mL). Spinal magnetic resonance imaging revealed sporadic T2WI hyperintensities in the thoracic cord below Th2, suggesting myelitis (Fig. 2a). Nerve conduction study suggested demyelination in nerve roots. Drug lymphocyte stimulation test showed suspicious for positive to nivolumab.

Finally, he was diagnosed as having a myeloradiculoneuropathy caused by nivolumab. He received two cycles of steroid‐pulse therapy (methylprednisolone 1000 mg/day for 3 days intravenously) from days 106 and 118 after the first nivolumab administration, respectively. After the treatment, his symptoms gradually improved, and he became able to walk on his own without any side effect of steroid‐pulse therapy. Results of the follow‐up lumbar puncture showed almost normal findings and abnormal signals below Th2 level on magnetic resonance imaging (T2WI) disappeared (Fig. 2b).

**Fig. 3:**
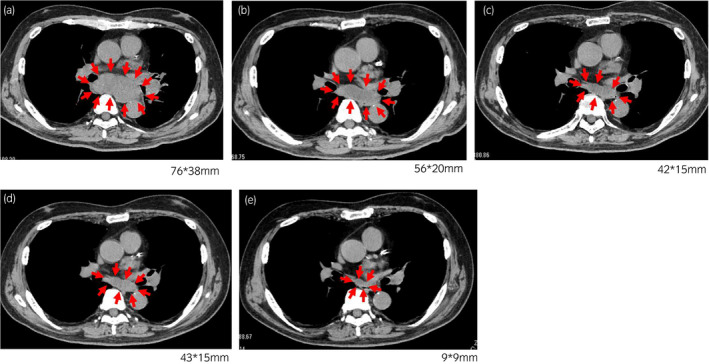
Computed tomography scan images demonstrate a decrease in size of mediastinum lymph nodes and lung metastases after three cycles of infusions of nivolumab. The size of mediastinum lymph node metastasis is shown in each figure. (a) Before treatment with nivolumab. (b) Just after three cycles of nivolumab administration. (c) Six months after last nivolumab administration. (d) One year after last nivolumab administration. (e) Two years after last nivolumab administration.

After only three cycles of nivolumab administration, the mediastinum lymph node metastasis began to shrink without any additional treatment (Fig. 3). The size of the metastatic lesion, which was 76 × 38 mm before treatment, was reduced to 56 × 20 mm just after the third administration and 42 × 15 mm at 6 months. Moreover, 2 years after the treatment with nivolumab, the size of lymph node metastasis has further shrunk to less than 10 mm, and lung metastasis has completely disappeared without any new lesions (CR based on Response Evaluation Criteria in Solid Tumors version 1.1).

## Discussion

Nivolumab is widely recognized as a standard second‐line treatment following antiangiogenic drugs for advanced RCC.^1^, ^2^, ^3^, ^4^ However, the underlying CheckMate 025 trial was focused for patients with clear cell carcinoma components, excluding non‐clear cell carcinoma.^5^ Therefore, the efficacy of nivolumab for non‐ccRCC including PRCC remains an important clinical issue.

Among few reports regarding the activity of nivolumab in patients with non‐ccRCC, Koshkin *et al*. reported the objective response rate was 20% in total from multicenter retrospective analysis.^7^ In another meta‐analysis of retrospective studies, the objective response rate was 21.6% and 8.8% achieved CR in non‐ccRCC.^8^ Nivolumab was effective in cases with unclassified, PRCC type 1, and rhabdoid variant.

There are a few case reports that nivolumab was effective for PRCC type 2.^9^, ^10^, ^11^, ^12^ As a noteworthy case, nivolumab showed a remarkable therapeutic effect without any irAEs in a patient with metastatic PRCC type 2, even in the setting of third‐line treatment after sunitinib and pazopanib.^10^ In contrast, the latest literature also reports combined immunotherapy of nivolumab plus ipilimumab shows the extremely lower efficacy in PRCC compared to clear cell carcinoma.^13^

The treatment with ICIs is associated with a wide spectrum of irAEs, but neurological symptoms are rare. In the review article, neurological irAEs related to central nerve system in 43 cases and peripheral nerve system in 82 cases were reported.^14^ Of these, only two cases were with myelitis, but no case of myeloradiculoneuropathy has been reported. The treatment for neurological irAEs included oral or intravenous steroids, intravenous immunoglobulins, plasma exchange, and other immunosuppressive drugs. But, complete recovery rate was quite low, only 37.2% of central nerve and 14.6% of peripheral nerve symptoms.

As a recent noteworthy topic, correlation between irAE and efficacy has been reported in several studies on malignant tumors including RCC.^15^, ^16^, ^17^ Moreover, durable response of nivolumab has been reported in patients with metastatic RCC.^18^

In our case, only three cycles of nivolumab led to a durable and CR in the patient with metastatic PRCC type 2. This is the first case of myeloradiculoneuropathy as irAE after the treatment with ICI, which was completely recovered by steroid‐pulse therapy. The limitation of this report is that the results were derived only from a single case and allergic reactions cannot be completely ruled out as a cause of myeloradiculoneuropathy.

## Conclusions

ICIs including nivolumab may be a promising treatment option for non‐ccRCC such as PRCC. Development of biomarkers useful for treatment selection is highly expected.

## Conflict of interest

The authors declare no conflict of interest.
